# Management of ureteral stenting for postrenal failure during pregnancy after ureteral reimplantation: a case report

**DOI:** 10.1186/s12884-016-0855-6

**Published:** 2016-04-01

**Authors:** Yutaka Yoneoka, Shoji Kaku, Shunichiro Tsuji, Hiroto Yamashita, Takashi Inoue, Fuminori Kimura, Takashi Murakami

**Affiliations:** Department of Obstetrics and Gynecology, National Hospital Organization Higashi-Ohmi General Medical Center, 255 Gochi-cho, Higashiohmi, Shiga 527-0044 Japan; Department of Obstetrics and Gynecology, Shiga University of Medical Science, Seta tsukinowa-cho, Otsu, Shiga 520-2192 Japan; Department of Urology, Kohka Public Hospital, 1256 Minakuchichomatsuo, kohka, Shiga 528-0074 Japan

**Keywords:** Ureteral stenting, Pregnancy, Ureteral reimplantation, Postrenal failure

## Abstract

**Background:**

Vesicoureteral reflux is thought to predispose to urinary tract infection and renal scarring, and ureteral reimplantation in childhood remains the gold standard for its treatment. It has been reported that the risk of postrenal failure during pregnancy is increased among women with Politano-Leadbetter ureteral reimplantation. In previous case reports on patients with progressive hydronephrosis and renal failure during pregnancy after ureteral reimplantation, percutaneous nephrostomy was always required, so there has been no information about the clinical management of such patients by ureteral stenting. Here we report a patient with a history of bilateral ureteral reimplantation, in whom severe hydronephrosis during pregnancy was managed with ureteral stents.

**Case presentation:**

A primigravida with severe hydronephrosis was referred to us at 29 weeks of gestation. Bilateral Politano-Leadbetter ureteral reimplantation had been performed at the age of 3 years. She was hospitalized immediately, and bilateral ureteral stents were successfully inserted. Post-obstructive diuresis occurred after the stents were placed. Urinary tract infection developed after removal of the urethral catheter 1 week later, but responded to antibiotic therapy and catheter replacement. Labor was induced at 39 weeks of gestation, with vaginal delivery of a healthy male infant. Both stents were found to have spontaneously migrated into the urethra after delivery. Repeat stenting under spinal anesthesia was required to improve postpartum symptoms of back pain and fever. Right distal ureteral obstruction persisted at 6 months after delivery and repeat ureteral reimplantation is planned.

**Conclusions:**

General obstetricians will not necessarily pay attention to a history of Politano-Leadbetter ureteral reimplantation, but these patients should undergo careful monitoring of renal function and urinary tract morphology during perinatal care. In the present case, ureteral stenting was effective for postrenal failure during pregnancy after ureteral reimplantation. If ureteral stenting is selected, attention should be paid to post-obstructive diuresis, infection, and stent migration.

## Background

In patients with vesicoureteral reflux (VUR), retrograde flow of urine from the bladder to the kidneys occurs due to failure of the ureterovesical valve mechanism. VUR is thought to predispose to urinary tract infection and renal scarring, with management involving both surgical correction and medical treatment. Ureteral reimplantation remains the gold standard, with common procedures being the Politano-Leadbetter technique and the Cohen technique.

Little information is available regarding the long-term outcome of antireflux surgery. It has been reported that women with a history of Politano-Leadbetter ureteral reimplantation have an increased risk of postrenal failure during pregnancy secondary to narrowing of the distal ureters, and all reported patients have required nephrostomy to achieve a good obstetric outcome [1–4].

Here we report a woman with Politano-Leadbetter ureteral reimplantation for VUR, in whom ureteral stenting rather than nephrostomy was performed to successfully manage severe hydronephrosis during pregnancy. The possible complications of ureteral stenting are also described.

## Case presentation

A 24-year-old primigravida presented to our hospital at 29 weeks of gestation. She had undergone bilateral ureteral reimplantation by the Politano-Leadbetter technique at the age of 3 years for vesicoureteral reflux and her postoperative course had been uneventful. However, she had mild back pain at presentation, and abdominal ultrasound revealed severe bilateral hydronephrosis. Blood pressure was 120/80 mmHg and serum creatinine was increased to 3.57 mg/dl. We diagnosed acute postrenal failure and hospitalized her immediately. A double-pigtail ureteral stent (4.7 French) was placed into each ureter by our senior urologist under ultrasound guidance without using fluoroscopy.

**Fig. 1:**
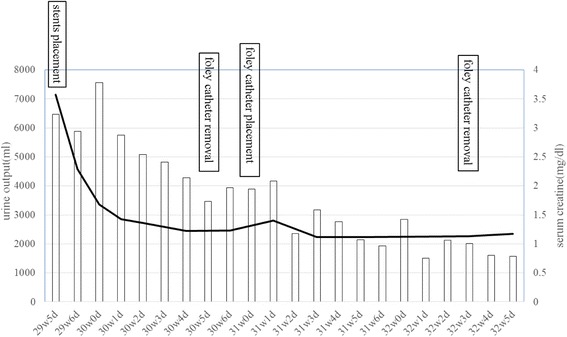
Course of daily urine output and serum creatine after ureteral stenting

Because daily urine output exceeded 6 L after ureteral stenting, a urethral catheter was inserted and saline was infused intravenously to prevent dehydration. Serum creatinine rapidly decreased to 1.1 mg/dl and hydronephrosis improved, so the urethral catheter was removed 7 days after ureteral stenting (Fig. 1). Subsequently, the patient developed high fever and severe back pain. Laboratory data and urine culture revealed evidence of pyelonephritis, with a white blood cell count of 13.1 × 10^3^/μL and C-reactive protein of 11.34 mg/dl. Streptococcus agalactiae was isolated from a urine specimen. Another urethral catheter was inserted and ceftriaxone sodium (2 g/day) was administered intravenously for 6 days, after which her symptoms resolved. The urethral catheter was removed again when urine output decreased. Because there was no longer any clinical or laboratory evidence of infection, she was discharged at 33 weeks of gestation. Signs or symptoms of preterm labor, such as shortening of the cervix or frequent uterine contractions, did not occur during hospitalization.

The patient was re-admitted at 39 weeks with gestational hypertension. Her systolic blood pressure was persistently higher than 140 mmHg, although no proteinuria was detected. After induction, she delivered a boy weighing 3140 g. The Apgar score was 9 at 1 min and 10 at 5 min.

She complained of back pain and fever on the day after delivery, and it was found that both ureteral stents had migrated into the urethra. Her symptoms subsided after stents were placed into the bilateral ureters again under spinal anesthesia. The patient was discharged two weeks after delivery. An urologist removed both stents at 6 months after delivery, but distal obstruction of the right ureter was noted and a stent was inserted on the right again. She is scheduled to undergo repeat bilateral ureteral reimplantation.

## Conclusions

A search of the English literature in PubMed using key words including “vesicoureteral reflux”, “ureteral reimplantation”, and “pregnancy” identified seven women with postrenal failure during pregnancy after undergoing ureteral reimplantation in childhood. Six of the seven women were managed by nephrostomy [Table 1].　　Laverson et al. [3] reported a patient with bilateral ureteral stenting, in whom they could not place ureteral catheters after stent removal due to edema at the ureteral orifices. Cesarean section was performed at 30 weeks of gestation and the baby died. This patient was treated with a right ureteral stent and left nephrostomy during her next pregnancy, resulting in delivery by repeat cesarean section at term. It seems to be difficult to insert ureteral catheters in these patients, although ureteral stenting is not uncommon in pregnant women [5]. In our patient, full term delivery was achieved after bilateral placement of double-pigtail ureteral stents to control hydronephrosis secondary to Politano-Leadbetter reimplantation for VUR.

**Table 1:** Cases of postrenal failure during pregnancy after Politano-Leadbetter ureteral reimplantation in childhood

Case No.	Reference	Presentation (weeks)	Treatment	Perinatal outcome	Reoperation after delivery	Subsequent pregnancy
1	[3]	28	bilat ureteral stent failed ureteral catheter replacement	C/S at 30 weeks neonatal death	no	rt ureteral catheter and lt nephrostomy at 25 weeks
2	[2]	30	lt nephrostomy	VD at full term	no	-
3	[1]	30	lt nephrostomy	VD at 33 weeks	no	lt nephrostomy at 32 weeks
4	[1]	20	rt nephrostomy	VD at 38 weeks	yes	bilat nephrostomy at 29 weeks
5	[1]	15	bilat nephrostomy	VD at full term	yes	no treatment for 2 uneventful pregnancies
6	[1]	30	rt nephrostomy	VD at 35 weeks	no	conservative treatment
7	[4]	25	lt nephrostomy	VD at 39 weeks	yes	-
8	present case	29	bilat ureteral stent	VD at 39 weeks	scheduled for reoperation	-

*C/S* cesarean section, *VD* vaginal delivery

However, several problems had to be overcome.

Post-obstructive diuresis is a dramatic increase of urine output after the relief of urinary tract obstruction and tends to be more severe in patients with chronic obstruction [6]. In our patient, diuresis began soon after ureteral stenting. In 2 of the reported patients, post-obstructive diuresis occurred after percutaneous nephrostomy at 30 weeks of gestation and after cesarean delivery at 30 weeks [1, 3]. In our patient, renal failure may have gradually developed from early pregnancy, although symptoms were only noted shortly before 29 weeks of gestation. Post-obstructive diuresis usually persists until a euvolemic state is reached and it is necessary to provide intravenous saline to avoid dehydration, hypotension, and electrolyte abnormalities, as was done in our patient.

One of the main complications associated with ureteral stents is urinary tract infection, which often occurs several weeks after stenting because of bacterial colonization [7]. Our patient developed symptoms of urinary tract infection accompanied by a strong inflammatory response and positive urine culture soon after the removal of the urethral catheter at 1 week after ureteral stent placement. Backflow of urine from the bladder into the kidneys may have occurred through the stents and resulted in infection because urine output was still high after removal of the urethral catheter, leading to rapid elevation of the intravesical pressure. Her symptoms subsided and laboratory findings improved after another urethral catheter was inserted and antibiotic therapy was provided. There was no recurrence of symptoms when the urethral catheter was later removed again, probably because urine output was much smaller. Our experience with this patient suggests that the urethral catheter should only be removed after urine output is close to normal.

Stent migration is an uncommon complication that is related to inadequate positioning of the lip of the stent [8]. In our patient, bilateral stent migration was noted after delivery, although there were no problems during pregnancy, suggesting that migration may be more likely during delivery because of uterine contraction and involution. It is difficult to predict or prevent stent migration, so it is important to be aware of this possibility and carefully observe the patient and the stent position.

According to previous reports, ureteral obstruction is usually alleviated at least 4 weeks after delivery [1, 2]. It may be relevant that physiological changes of pregnancy revert to normal about 6 weeks after delivery. However, ureteral obstruction was not relieved in some patients [1–4] and surgery was required. The course of subsequent pregnancy also varies. The patient reported by Laverson et al. did not receive surgical reimplantation after her first pregnancy and hydronephrosis recurred during the next pregnancy [3]. The first patient reported by Mor et al. refused repeat reimplantation and hydronephrosis recurred during her second pregnancy. Their second patient received repeat ureteral reimplantation by the Cohen method 3 years before her next pregnancy, but she still required bilateral nephrostomy due to hydronephrosis, hypertension, and azotemia at 29 weeks of gestation. Their third patient had repeat reimplantation and subsequently experienced two uneventful pregnancies without urological complications. In their fourth case, recurrent renal infection was a problem during the first pregnancy and right percutaneous nephrostomy was performed during the second pregnancy, but conservative treatment was sufficient for the third pregnancy [1]. In these patients, ureteral obstruction seems to be a transient phenomenon during pregnancy and is impossible to predict. Our patient is scheduled for reimplantation surgery before her next pregnancy. Even so, she should be carefully monitored for hydronephrosis by abdominal ultrasound and renal function tests, as well as symptoms such as back pain, during her next pregnancy.

In conclusion, Politano-Leadbetter reimplantation for VUR is associated with an increased risk of renal failure during pregnancy due to distal ureteral obstruction. Based on our experience, ureteral stenting is one of the therapeutic options. If it is possible to insert stents, attention should be paid to problems such as post-obstructive diuresis, urinary tract infection, and stent migration after delivery.

## Consent

Written informed consent was obtained from the patient for publication of this Case report and any accompanying images. A copy of the written consent is available for reviewer by the Editor of this journal.

## Abbreviation

VUR: vesicoureteral reflux
